# In-Season Concussion Symptom Reporting in Male and Female Collegiate Rugby Athletes

**DOI:** 10.1089/neur.2021.0050

**Published:** 2021-11-16

**Authors:** Emily E. Kieffer, Per Gunnar Brolinson, Arthur E. Maerlender, Eric P. Smith, Steven Rowson

**Affiliations:** ^1^ School of Biomedical Engineering and Sciences and Virginia Tech, Blacksburg, Virginia, USA.; ^2^ Edward Via College of Osteopathic Medicine, Blacksburg, Virginia, USA.; ^3^ Center for Brain, Biology and Behavior, University of Nebraska at Lincoln, Lincoln, Nebraska, USA.; ^4^ Department of Statistics, Virginia Tech, Blacksburg, Virginia, USA.

**Keywords:** concussion, sex-specific, subconcussion, symptoms

## Abstract

Symptom inventories are generally only collected after a suspected concussion, but regular in-season monitoring may allude to clinical symptoms associated with repetitive subconcussive impacts and potential undiagnosed concussions. Despite sex-specific differences in symptom presentation and outcome of concussion, no return-to-play protocol takes sex into account. The objective of this study was to monitor a cohort of contact-sport athletes and compare the frequency and severity of in-season concussion-like symptom reporting between sexes. Graded symptom checklists from 144 female and 104 male athlete-seasons were administered weekly to quantify the effect of subconcussive impacts on frequency and severity of in-season symptom reporting. In-season, mean symptom severity score (SSS) (*p* = 0.026, mean difference of 1.8), mean number of symptoms (*p* = 0.044, mean difference of 0.9), max SSS (*p* < 0.001, mean difference of 19.2), and max number of symptoms (*p* < 0.001, mean difference of 6.8) were higher in the females. The females' survey results showed differences between elevated and concussed SSS (*p* < 0.005, mean difference of 28.1) and number of symptoms reported (*p* = 0.001, mean difference of 6.6). The males did not have a difference in SSS (*p* = 0.97, mean difference of 1.12) nor in number of symptoms (*p* = 0.35, mean difference of 1.96) from elevated to concussed athletes. Rugby players report concussion-like symptoms in the absence of a diagnosed concussion in-season. Female athletes reported elevated symptom frequencies with greater severities than the males, but both sexes reported considerable levels throughout the season.

## Introduction

Concussion diagnosis has taken a multi-pronged approach to include a consideration of clinical history, acute sideline evaluation, symptom assessment, detailed neurological evaluation, and neurophysiological testing.^1^ Diagnosis is complicated by athletes' tendency to hide or underreport symptoms to decrease return-to-play time.^2^ Some studies have indicated that 30–50% of concussions go unreported.^2,3^ Athletes who do not report concussion symptoms immediately and continue to participate in activities may be at higher risk for longer recoveries and sustain post-concussion symptoms longer.^4^

Variation in presentation and outcome of concussion by sex has been established previously.^5–10^ Beyond physiological explanations, systematic differences in symptom reporting have been suggested, because females report more concussions and greater severities than males.^5,11,12^ Previous studies have noted that females report higher symptoms at baseline^1,13^ as well as post-injury.^7,13^ Females have also exhibited a greater cognitive change post-concussion and more variation in cognitive assessment performance than their male counterparts.^14^ Despite these differences, no return-to-play protocol takes sex into account.^7,15,16^

Graded symptom checklists are used commonly in concussion diagnosis and have been shown to differentiate between concussed and non-concussed athletes with a sensitivity of 64%–89% and a specificity of 91–100%.^17–21^ In combination with neuropsychological assessments, symptom resolution generally is the guideline for return-to-play for the athlete.^15^ Regular monitoring of in-season symptoms would help researchers understand the presentation of subconcussive impacts that are below typical diagnostic thresholds and help identify cases of elevated symptoms in-season and potential undiagnosed concussions.

This study's objective was to monitor and compare in-season concussion symptom reporting between sexes. We studied a cohort of collegiate rugby players because females and males play by the same rules and routinely experience head impacts.^22,23^ We hypothesize that rugby athletes routinely experience concussion-like symptoms, in the absence of a diagnosed concussion, throughout a season and that symptom presentation is sex-specific. Although we cannot attribute all symptom presentation to head impact exposure, because these symptoms are not unique to concussions and other factors could cause similar presentation, we were interested in the severity and frequency of concussion-like symptom presence in the context of a collision sport.

## Methods

### Subjects

Men's and women's club rugby teams participated in this study from Spring 2018 – Spring 2020. The Spring 2020 season was ended abruptly with COVID-19. Written informed consent was obtained from each participant in accordance with the ethical guidelines of the Institutional Review Board (IRB). There were 58 females and 57 males who participated over the four seasons (Spring and Fall), with many participating in multiple seasons. A total of 144 female-seasons (age: 20.5 ± 1.3 years, height: 1.66 ± 0.08 m, weight: 73.3 ± 17.1 kg) and 107 male-seasons (age: 20.6 ± 1.3 years, height: 1.80 ± 0.08 m, weight: 89.4 ± 15.6 kg) participated. Athletes participated in a mix of rugby union and rugby sevens.

### Symptom checklists

Before starting the season and at the beginning of each week in-season, athletes were e-mailed a survey that included a Graded Symptom Checklist (GSC) and an open-ended question. The GSC consisted of 27 symptoms that athletes graded on a scale of 0–6, with 0 indicating a symptom was not present and 6 representing the most severe presentation (Table 1).^24^ Studies have validated the use of GSCs with measures of balance and neurocognitive function and with the presentation of post-traumatic headache.^19,25^ The total symptom frequency score (maximum of 27) and the aggregate score, computed as the Symptom Severity Score (SSS) (maximum of 162), were quantified by athlete per week.

**Table 1.** List of 27 Concussion Symptoms in the Graded Symptom Checklist that Subjects Grade on a Scale of 0 (None) to 6 (Most Severe)

Blurred Vision	Loss of Consciousness	Sadness
Dizziness	Loss of Orientation	Seeing Stars
Drowsiness	Memory Problems	Sensitivity to Light
Easily Distracted	Nauseous	Sensitivity to Noise
Fatigue	Nervousness	Sleep Disturbances
Feeling “In a Fog”	Personality Changes	Sleeping More than Usual
Feeling “Slowed Down”	Poor Balance/Coordination	Unusually Emotional
Headache	Poor Concentration	Vacant Stares/Glassy Eyes
Irritability	Ringing in the Ears	Vomiting

Total scores are determined for the numbers of symptoms reported (maximum of 27) and total Symptom Severity Score (maximum of 162).

The open-ended question asked athletes to report anything notable from the previous week that could explain their symptoms (such as a hard head impact, sickness, stress, etc.). Surveys were validated with Fall 2019 Sports Concussion Assessment Tool 5 (SCAT5) baseline data administered through Virginia Tech Club Sports.^26^ The SSS and number of symptoms reported were paired for 31 athlete-seasons from Fall 2019 and correlated with the Pearson coefficient (r).

### Statistical analysis

Any survey that mentioned a reason other than head impact during play for their symptom presentation was excluded from the analysis. Some of these reasons included illness, car accident, schoolwork, musculoskeletal injury, premenstrual syndrome, and mental health. Surveys from athletes who did not participate the week leading up to that survey were also excluded. Surveys were grouped by athlete-seasons and summarized to identify any symptom presentations (SSS >0) during baseline and in-season. A McNemar chi-square test was used to determine whether there was a relationship between symptom presentation at the two time points: baseline and in-season. Data were then summarized at the athlete level (combining multiple seasons worth of data for some athletes).

The mean and maximum number of symptoms and SSS were quantified for baseline and in-season for each athlete. The median and interquartile ranges (IQR) were computed for each time point and sex. The data were right skewed, so a paired Wilcoxon signed-rank test was used to determine whether athletes reported more symptoms in-season compared with baseline within sex. Effect size and precision were estimated. To determine whether there were differences in reporting between sexes at the two time points, a Wilcoxon rank-sum test was implemented. Effect size and precision were estimated.

We chose the maximum baseline SSS for each sex to represent a threshold for “elevated” symptom presentation in-season. The threshold for both sexes was 11. The proportion of athlete-seasons that reported elevated symptoms was calculated. We also quantified the proportion of those athlete-seasons that reported recurring elevated symptoms and the number of weeks for which they were reported. We defined recurring elevated symptoms as occurring when an athlete reported more than one week of elevated symptom presentation within a season, not necessarily in consecutive weeks. A chi-square test was used to compare the proportions of elevated athlete-seasons per sex. A Fisher exact test was used to compare the proportions of recurrent elevated athlete-seasons per sex.

To compare elevated SSS by sex, the elevated surveys' SSS median and IQR were analyzed with Wilcoxon tests, and mean differences were compared to estimate the effect size. The same was performed for the median and IQR of the number of symptoms reported. The mean and maximum values of each symptom were computed per sex. We defined an individual SSS greater than 2 (>2) as at least moderate severity and computed the proportion of surveys that reported a given symptom with at least moderate severity and compared the differences in proportions between sexes.

We wanted to identify similarities in symptom presentation between subconcussion levels and diagnosed concussion levels. To compare reporting between athletes with a clinical diagnosis and those who reported elevated symptoms, a Wilcoxon rank-sum test was used. The effect size and precision were estimated with a Welch two-sample *t* test. The elevated surveys were from athlete-seasons in which athletes did not sustain a concussion so that post-concussion surveys did not influence the comparison. For all statistics, α = 0.05 was used.

## Results

During the 144 female-seasons, 1440 of 1751 surveys (82%) were returned. During the 107 male-seasons, 793 of 938 surveys (85%) were returned. The 123 surveys from females and 66 surveys from males indicated confounding causes of symptoms and were excluded from the analysis. There were 1317 surveys from females and 727 surveys from males included in the analysis. There was no baseline data for Spring 2018 or Fall 2018 because the IRB was not approved before the season started. So, baseline data were from Spring 2019–Spring 2020 only. Fall 2019 baseline SSS and number of symptoms are significantly correlated with SCAT5 results (SSS: r = 0.70, *p* < 0.001; number of symptoms: r = 0.58, *p* < 0.001). Of study participants, 10 females and four males had a diagnosis of concussion. On average, they completed their survey two days post-concussion (range = 0–6).

### Overall presence of symptoms

**FIG. 1.**
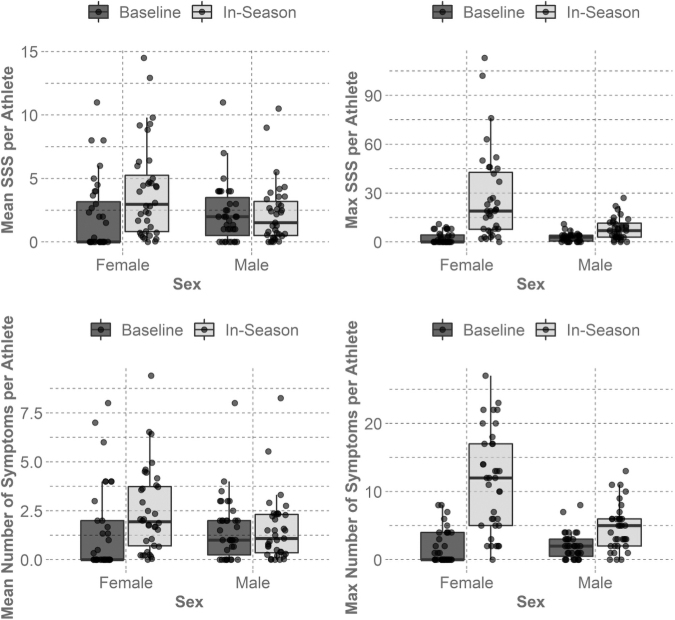
Box plots and points for mean and max Symptom Severity Score (SSS) and number of symptoms for males and females for paired baseline and in-season surveys. The median of the mean and max SSS and number of symptoms were higher in-season than those at baseline for the females, but were much more similar in magnitude for the males. These data are summarized by athlete per time point.

When comparing athlete-season symptom presentation between the two time points, the McNemar chi-square test suggested a relationship between symptom presentation (SSS >0) at baseline and in-season (*p* < 0.001). If an athlete reported symptoms at baseline, he or she was more likely to report symptoms in-season. Table 2 and Figure 1 show the distribution of symptom presentation between time points for each sex.

**Table 2.** Summary of Symptom Presentation Reported from Paired Baseline and In-Season Time Points for Male and Female Athletes

	Time point	Athlete count	SSS median of the mean [IQR]	SSS median of the max	# Symptoms median [IQR]	# Symptoms median of the max
Females	Baseline	36	0 [0, 3.2]	0	0 [0, 2]	0
	In-Season	36	3 [0.8, 5.2]	19	1.9 [0.7, 3.7]	12
Males	Baseline	35	2 [0.5, 3.5]	3	1 [0.3, 2]	2
	In-Season	35	1.5 [0.5, 3.2]	7	1.1 [0.4, 2.3]	5

Males reported a higher mean Symptom Severity Score (SSS) at baseline, but females reported higher SSS and number of symptoms in-season. The median scores of 0s suggest many surveys had reports of low symptom presentation. The mean and max SSS and number of symptoms were calculated for each athlete, and then the median and interquartle range (IQR) of the groups were summarized.

The female athletes reported higher mean SSS (*p* = 0.001, mean difference of 2.1 [95% confidence interval (CI): 0.9, 3.3]), number of symptoms (*p* = 0.003, mean difference of 1.1 [95% CI: 0.3, 1.9]), higher max SSS (*p* < 0.001, mean difference of 24.7 [95% CI: 15.4, 33.9]), and higher max number of symptoms (*p* < 0.001, mean difference of 9.7 [95% CI: 7.1, 12.3]) in-season compared with their baseline time point. The male athletes reported similar mean in-season SSS (*p* = 0.645, mean difference of 0.02 [95% CI: -0.7, 0.7]), number of symptoms (*p* = 0.6671, mean difference of 0.041 [95% CI: -0.4, 0.5]), higher max SSS (*p* < 0.001, mean difference of 5.2 [95% CI: 3.2, 7.3]), and higher max number of symptoms (*p* < 0.001, mean difference of 2.7 [95% CI: 1.7, 3.6]) compared with their baseline time point.

At baseline, the mean SSS for females was not higher than for the males (*p* = 0.151, mean difference of 0.3 [95% CI: -1.5, 0.9]), nor was the mean number of symptoms reported per athlete (*p* = 0.102, mean difference of 0.2 [95% CI: -1.1, 0.7]), max SSS (*p* = 0.204, mean difference of 0.2 [95% CI: -1.7, 1.2]), nor max number of symptoms reported (*p* = 0.163, mean difference of 0.3 [95% CI: -1.3, 0.8]). In-season, however, mean SSS (*p* = 0.026, mean difference of 1.8 [95% CI: 0.3, 3.2]), mean number of symptoms reported (*p* = 0.044, mean difference of 0.9 [95% CI: 0.02, 1.86]), max SSS (*p* < 0.001, mean difference of 19.2 [95% CI: 9.7, 28.7]), and max number of symptoms reported (*p* < 0.001, mean difference of 6.8 [95% CI: 4.0, 9.5]) were higher in the females.

### Elevated symptom presence

At some point during the season, 59 (41.5%) female athletes and 15 (14.7%) male athletes reported elevated symptoms (SSS ≥11). The proportion of females who reported elevated symptoms was higher than that of males (chi-square test for proportions, *p* < 0.001, 95% CI: 0.15, 0.38). Thirty (50.8%) female athletes and four (26.7%) male athletes reported recurrent elevated symptoms. The Fisher exact test was used to determine whether the reporting of recurrent elevated symptoms was related to sex (*p* = 0.146, 95% CI: 0.074, 1.38).

For athletes who reported recurrent symptoms, we quantified the number of surveys on which they reported elevated symptoms, and then compared those values between sexes. On average, 3.1 ± 1.8 surveys completed by this subset of females (29.5% ± 14.3 of their surveys) and 3.3 ± 1.9 surveys completed by this subset of males (47.8% ± 18.5) reported elevated symptoms.

**FIG. 2.**
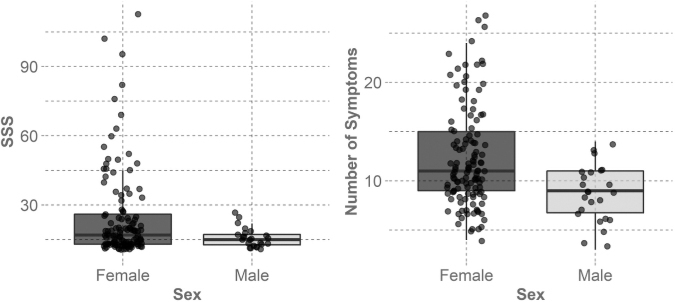
Box plots with data points for Symptom Severity Score (SSS) and symptom severity for elevated surveys. Outliers from the box plots were not pictured so that those data points were not plotted twice. Although the central tendencies do not seem to vary greatly between females and males, the upper end of both SSS and the number of symptoms reported is higher in females, highlighting a difference in distribution. The males were more evenly distributed across the scores while more females tended to report on the higher end, skewing the distribution toward the right.

The severity of elevated symptoms differed by sex. Table 3 summarizes elevated surveys per sex, and Figure 2 shows that the central tendencies were similar, but deviate at the higher end of the IQR. The difference between the median SSS is 2, compared with the difference at the 75th percentile, where the difference is 9. The SSS showed evidence of a difference between sexes, with a mean difference of 8.9 (95% CI: 5.0, 12.8, *p* = 0.044). The number of symptoms reported showed more evidence of difference between sexes, with a mean difference of 3.6 (95% CI: 2.1, 5.1, *p* = 0.0012).

**Table 3.** Number of In-Season Surveys Collected in Total and Those that Reported Elevated Symptom Severity Score per Sex

	In-Season	Elevated (SSS ≥11)	SSS median [IQR]	# Symptoms median [IQR]
Females	1251	121 (9.67%)	17 [13, 26]	11 [9, 15]
Males	674	24 (3.56%)	15 [12.8, 17.3]	9 [6.8, 11]

A higher proportion of female surveys reported elevated symptoms. The females' surveys also had a higher median SSS and number of symptoms reported. SSS, symptom severity score; IQR, interquartile range.

**FIG. 3.**
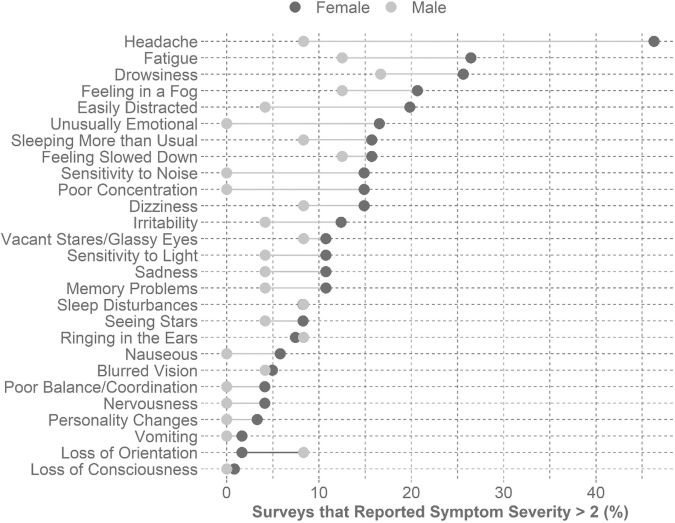
Plot of differences in the percentage of elevated surveys that report the given symptom with moderate or greater severity (>2) between sexes. A higher proportion of females reported all symptoms with a severity greater than 2 except loss of orientation (-6.7%), ringing in the ears (-0.9%), and sleep disturbances (-0.1%). The other symptoms' mean difference was 8.5%, ranging between 0.8% and 16.5%, except for headache (37.9%).

The highest reported mean severity for a symptom for females was headache, followed by fatigue and drowsiness. The highest mean symptom score for the males was drowsiness, followed by fatigue and feeling in a fog. The females reported higher symptom severity overall (Fig. 3). Of the 27 symptoms, females reported the maximum severity for 17 symptoms and the males only reported maximum severity for one symptom.

### Concussion symptom presentation

There were 14 diagnosed concussions in the dataset. The median and max SSS and number of symptoms for the athletes with the diagnosis and elevated surveys are summarized in Table 4. The females' survey results showed differences between elevated and concussed SSS (*p* < 0.005, mean difference of 28.1 [95% CI: 3.0, 53.2]) and number of symptoms reported (*p* = 0.001, mean difference of 6.6 [95% CI: 2.2, 10.9]). The males, however, did not have a difference in SSS (*p* = 0.97, mean difference of 1.12 [95% CI: -10.6, 12.9]) nor in number of symptoms reported (*p* = 0.35, mean difference of 1.96 [95% CI: -3.9, 7.8]).

**Table 4.** Summary of Symptom Presentation in Athletes who Received a Concussion Diagnosis from Clinical Staff and Those Who Reported Elevated Symptom Severity Score

Sex	Category	Survey count	SSS median (max)	# of symptoms median (max)
Female	Concussion	10	46 (113)	17.5 (27)
	Subconcussion	92	16 (102)	10 (26)
Male	Concussion	4	15 (27)	10.5 (14)
	Subconcussion	21	15 (25)	9 (13)

Two female athletes sustained concussions twice; the rest of the concussions are from unique athletes. Females report more symptoms with a higher severity than their male counterparts post-concussion. SSS, symptom severity score.

There were nine additional athletes with suspected concussions—i.e., they reported a suspected concussion to research staff but not to medical personnel. These suspected concussions had a median SSS of 25 (max = 48) and a median number of symptoms of 13 (max = 21). They were included in the subconcussion dataset, because they were not clinically diagnosed and had a SSS ≥11.

## Discussion

The rugby players commonly reported concussion symptoms in-season in the absence of diagnosed concussion, but at lower severities than those associated with a diagnosed concussion.^20,25^ Previous work has identified cognitive and neuroimaging changes in post-season testing in various sports, but in-season symptoms have not been monitored before.^27–38^ The elevated symptom cases we identified in our cohort may be the subconcussion injuries thought to cause the post-season changes previously measured. Sex-specific differences were also noted: females generally reported more symptoms with higher severity than their male counterparts. More females' surveys were categorized as elevated, and females more commonly reported symptoms of headache, anxiety, and mood.

While most athletes reported 0 symptoms at baseline, consistent with the literature,^39–41^ we observed that those who did report symptoms at baseline were more likely to report symptoms in-season, potentially indicating reporting tendencies. This pattern was stronger in the male athletes than the female athletes. Females reported higher in-season symptoms compared with their baseline and higher than males in-season. These results are consistent with other studies that indicate females report more baseline symptoms with higher severity.^1,7^

To identify a potential accumulation of symptoms, the number of weeks of recurrent elevated symptoms was quantified. The results did not vary significantly by sex, which is of note because previous work has identified post-concussion symptoms lasting longer in female athletes than their male counterparts.^7,42,43^ Of the males who reported recurrent elevated symptoms in this study, their elevated surveys made up a higher percentage of their total surveys compared with the females. One explanation for this is that the male athletes were more likely to fill out a survey if they were experiencing symptoms. Another reason for the discrepancy may be because of the small sample size.

Headache has been the most frequently reported symptom in other studies,^10^ similar to the females in our study. Females have a higher frequency for pre-existing headache,^44^ which may explain a more frequent or severe reporting in-season and post-concussion.^45^ Other most commonly reported symptoms by both sexes with at least moderate severity included fatigue and drowsiness. Both could be more reflective of a cohort of collegiate subjects and not specific to contact-sport athletes. A higher percentage of surveys from female athletes reported symptoms with at least moderate severity, and females reported maximum severities for more symptoms than males (17 vs. 1). These sex-specific patterns could be a systematic difference in reporting or a difference in experiences; we cannot determine from these data. Previous work has shown that there is some evidence for a correlation between athletes' ability to identify concussion symptoms and their reporting tendencies.^46–48^

Previous symptom assessments in concussed collegiate football players have shown a median of 10 symptoms (of 22) with a median severity score of 21 (of 132), which are similar to the current study given the maximum possible scores of each survey.^42^ The female athletes showed differences between the subconcussive and concussive levels, but the males did not. This sex-specific difference could be a result of the males' concussion severities being lower than the females' or that males experience higher level subconcussive events in-season. Post-concussion symptoms present themselves on a different timeline for each individual, and those differences are likely highlighted. As each week is treated in isolation, we may have missed delayed presentation of symptoms.

Surveys that had mentioned confounding reasons for symptom presentation were excluded. The symptoms included in the symptom checklist, however, are not exclusive to concussions and could result from factors besides head impact exposure. For this study, we were seeking to understand patterns in presentation between male and female collegiate rugby players and understand that the results presented may not be specific to sport-related head impacts. Because we do not know the honesty in which athletes are completing their surveys, the analysis is limited in that sex-specific differences cannot explicitly be attributed to differences in reporting or differences in experience. Another limitation of the study is that there is no control group; we cannot compare the symptom presentation of the athletes in this study to a group of collegiate athletes in the absence of head impact exposure.

In this study, we have shown that rugby players report concussion-like symptoms in the absence of diagnosed concussion during the course of a season. We acknowledge that we cannot attribute all of the symptom presentation to head impact exposure. The implications of these symptom reports are unclear because they might represent undiagnosed concussions, subconcussive tissue changes, or transient subclinical effects. How they contribute to overt injury still needs to be determined.

Recent work from the Concussion Assessment, Research and Education consortium demonstrated a relationship between the amount of head impact exposure during the season and subsequent concussion.^49^ A relationship between measured head impact exposure and these “ambient” symptoms is of great interest and may offer insight into undiagnosed concussions. In this sample, female rugby players reported elevated symptom frequencies with greater severities than the males during the season. Both males and females, however, reported considerable levels of symptoms throughout the course of a season. Further strategies for addressing these in-season symptom responses should be considered.

## Abbreviations Used

CI: confidence interval
GSC: graded symptom checklist
IQR: interquartile range
SSS: symptom severity score
